# Antimicrobial susceptibility patterns of Ureaplasma species and Mycoplasma hominis in pregnant women

**DOI:** 10.1186/1471-2334-14-171

**Published:** 2014-03-28

**Authors:** Mathys J Redelinghuys, Marthie M Ehlers, Andries W Dreyer, Hennie A Lombaard, Marleen M Kock

**Affiliations:** 1 Department of Medical Microbiology, University of Pretoria, Pretoria, South Africa; 2 Department of Medical Microbiology, Tshwane Academic Division, National Health Laboratory Service, Pretoria, South Africa; 3 Department of Obstetrics and Gynaecology, University of Pretoria, Pretoria, South Africa

**Keywords:** Pregnant women, *Ureaplasma* spp., *Mycoplasma hominis*, Antimicrobial susceptibilities

## Abstract

**Background:**

Genital mycoplasmas colonise up to 80% of sexually mature women and may invade the amniotic cavity during pregnancy and cause complications. Tetracyclines and fluoroquinolones are contraindicated in pregnancy and erythromycin is often used to treat patients. However, increasing resistance to common antimicrobial agents is widely reported. The purpose of this study was to investigate antimicrobial susceptibility patterns of genital mycoplasmas in pregnant women.

**Methods:**

Self-collected vaginal swabs were obtained from 96 pregnant women attending an antenatal clinic in Gauteng, South Africa. Specimens were screened with the Mycofast Revolution assay for the presence of *Ureaplasma* species and *Mycoplasma hominis*. The antimicrobial susceptibility to levofloxacin, moxifloxacin, erythromycin, clindamycin and tetracycline were determined at various breakpoints. A multiplex polymerase chain reaction assay was used to speciate *Ureaplasma* positive specimens as either *U. parvum* or *U. urealyticum*.

**Results:**

Seventy-six percent (73/96) of specimens contained *Ureaplasma* spp., while 39.7% (29/73) of *Ureaplasma* positive specimens were also positive for *M. hominis*. Susceptibilities of *Ureaplasma* spp. to levofloxacin and moxifloxacin were 59% (26/44) and 98% (43/44) respectively. Mixed isolates (*Ureaplasma* species and *M. hominis*) were highly resistant to erythromycin and tetracycline (both 97% resistance). Resistance of *Ureaplasma* spp. to erythromycin was 80% (35/44) and tetracycline resistance was detected in 73% (32/44) of *Ureaplasma* spp. Speciation indicated that *U. parvum* was the predominant *Ureaplasma* spp. conferring antimicrobial resistance.

**Conclusions:**

Treatment options for genital mycoplasma infections are becoming limited. More elaborative studies are needed to elucidate the diverse antimicrobial susceptibility patterns found in this study when compared to similar studies. To prevent complications in pregnant women, the foetus and the neonate, routine screening for the presence of genital mycoplasmas is recommended. In addition, it is recommended that antimicrobial susceptibility patterns are determined.

## Background

*Mycoplasma hominis* and *Ureaplasma* spp., including *U. parvum* and *U. urealyticum,* are collectively known as genital mycoplasmas and are found in the vaginal milieu of up to 80% of pregnant and non-pregnant women [1]. The pathogenesis of genital mycoplasmas is still poorly understood. Damage related to genital mycoplasma infections might be the result of the induced immune- and inflammatory responses rather than the direct toxic effects of mycoplasma cellular components [2]. *Mycoplasma hominis* is specifically associated with conditions such as endometritis [3] and preterm birth [4]. Ureaplasmas are reported to be more prevalent than other mycoplasmas in the female urogenital tract, with *U. parvum* found more often than *U. urealyticum*[5]. During pregnancy, *Ureaplasma* spp. can cause chorioamnionitis, spontaneous abortion, stillbirth and preterm delivery [3]. Although the pathogenic role of *U. urealyticum* in urogenital tract infections is widely recognised [6-8], the role of *U. parvum* in these infections is not that well established [7]. Nonetheless, *U. parvum* might be present in bacterial loads leading to adverse pregnancy outcomes [9] and produce asymptomatic infections of the upper genital tract in women as frequently as *U. urealyticum*[7]. Out of the four *U. parvum* serovars (including serovars 1, 3, 6 and 14), serovars 3 and 14 have been isolated in more cases of genital tract infections than serovars 1 and 6 [10,11]. The greater virulence reported for *U. urealyticum* in some conditions might be attributed to its superior capability of acquiring genes horizontally [12].

Genital mycoplasmas display inherent resistance to beta-lactams and glycopeptides (e.g. vancomycin) because of the absence of a cell wall [13]. Although macrolides are often the drugs of choice for treating these infections, *M. hominis* is intrinsically resistant to the C14 and C15 macrolides (e.g. erythromycin and azithromycin) [14]. *Ureaplasma* species also have natural resistance to lincosamides (e.g. clindamycin) [15]. Observed resistance to macrolides is associated with mutations in the 23S rRNA gene [16,17], while resistance to tetracyclines is associated with the presence of the moveable *tet*(M) genetic element [18,19].

The administration of antimicrobial agents to pregnant women with preterm rupture of the membranes (PROM) may extend the gestation period and decrease the risks of associated complications and neonatal infections [20]. The antimicrobial agent of choice should be considered carefully, as some agents are teratogens - i.e. the agent can cause malformation or functional damage to an embryo or foetus or may have toxic effects on the neonate [21]. Macrolides are often used empirically [22] because of tetracyclines and fluoroquinolones being contraindicated in pregnancy [20,23]. However, the amniotic sac is not effectively penetrated by erythromycin and ureaplasmas are not eradicated from the vagina or cervix by this agent [13]. Newer macrolides (e.g. azithromycin and clarithromycin) allow for better tolerability and the once daily dosing benefit can increase compliance [13,24]. Treatment with azithromycin is equally successful compared to erythromycin but with fewer side effects [25,26].

To perpetuate the effective use of antimicrobial agents, the antimicrobial activities of such agents need to be monitored frequently. The Mycofast Revolution assay (ELiTech Diagnostic, France) is a commercially available assay that allows for detection, identification and antimicrobial-susceptibility testing of genital mycoplasmas within 48 hours [27]. However, identification with this assay is limited to *M. hominis* and the *Ureaplasma* genus [27]. The speciation of genital mycoplasmas can be achieved by the use of sensitive and rapid molecular methods, such as polymerase chain reaction (PCR) assays [28]. Speciation of bacteria may assist in elucidating the pathogenesis of specific medical conditions [29]. The purpose of this study was to investigate the antimicrobial susceptibility patterns of genital mycoplasmas in pregnant women attending antenatal care.

## Methods

This investigative study included pregnant women attending the antenatal and maternal and foetal unit (MAFU) clinics of a tertiary academic hospital in Pretoria, South Africa, from October 2012 to January 2013. Patients older than 18 years were included in the study. All women who participated gave written informed consent prior to commencement. Ethical approval was obtained from the Student Ethics Committee of the Faculty of Health Sciences, University of Pretoria (protocol number S6/2012) and preceded experimental work. Experimental work was conducted at the Department of Medical Microbiology, University of Pretoria.

Self-collected vaginal swabs (Copan Diagnostics, Inc, Italy) were obtained and tested for the presence of *Ureaplasma* species and *M. hominis*. Genital mycoplasmas were identified and enumerated and antimicrobial susceptibilities were determined with the Mycofast Revolution assay as indicated by the manufacturer. The swab was used to seed the UMMt transport medium of which 100 μl were added to the *U. urealyticum* (UU) and *M. hominis* (MH) wells of the Mycofast Revolution Screening tray. Additionally, 50 μl of MH supplement (S.Mh) were added to the MH well; the wells were covered with two drops of sterile mineral oil; and the tray was incubated (Vacutec, South Africa) at 37°C for 24 h. After 24 h of incubation, an orange or red colour change indicated the presence of *Ureaplasma* species and/or *M. hominis*. A yellow colour in the wells marked the absence of genital mycoplasmas. In the case of a positive screening test, the remainder of the seeded UMMt that was stored at 4°C to 8°C was used to inoculate the Complement Mycofast Revolution tray. One hundred microliters of UMMt medium were dispensed into wells 1 to 20 and 50 μl of S.Mh were dispersed into wells 6 and 7. All the wells were covered with two drops of sterile mineral oil. The tray was incubated (Vacutec, South Africa) at 37°C ± 1°C for 24 h (maximum 48 h in all cases) and the presence or absence of colour changes at defined breakpoints specified on the inoculation trays indicated resistance or susceptibility to each antimicrobial agent.

Genital mycoplasma strains were regarded as sensitive when growth was inhibited by the higher and lower critical concentrations of the antimicrobial agent. Genital mycoplasma strains were regarded as resistant when there was growth at the lower critical concentration of the antimicrobial agent but not the higher critical concentration. Strains were also regarded as resistant when there was growth at both the lower and higher critical concentrations of the antimicrobial agent. The specific breakpoints indicating susceptibility (S) or resistance (R) for *Ureaplasma* species were as follows [30]: levofloxacin S ≤2, R ≥4; moxifloxacin S ≤2; erythromycin S ≤8, R ≥16; and tetracycline S ≤1, R ≥2. The breakpoints for *M. hominis* are as follows: levofloxacin S ≤1, R ≥2; moxifloxacin S ≤0.25; clindamycin S ≤0.25, R ≥0.5; and tetracycline S ≤4, R ≥8.

A multiplex PCR (mPCR) assay was used to determine the respective *Ureaplasma* species detected with the Mycofast Revolution assay. This assay was performed as described by Stellrecht *et al*. [3] and was validated with reference strains ATCC27813 (*U. parvum*) and ATCC27619 (*U. urealyticum*). The multiple-banded antigen (MBA) gene of *Ureaplasma* served as the target gene. The amplified products were subjected to gel electrophoresis at 100 V for 1 h on a 2% (m/v) MetaPhor agarose gel (Lonza, USA) in 1X TBE buffer [45 mM Tris-borate (pH 8.0) (Sigma Chemical co, USA), 1 mM EDTA (Promega, Madison, USA)]. A 50 kb molecular marker (Fermentas, Thermo Scientific, USA) was used to identify band sizes, which were 403 bp and 448 bp in size for *U. parvum* and *U. urealyticum*, respectively [2].

The results were reported as percentages. A two-sample t-test between proportions was performed to determine whether there was a significant difference between the resistance of *Ureaplasma* species and mixed isolates to erythromycin and tetracycline.

## Results

The study population included 96 pregnant African women. The ages of the women were normally distributed and ranged from 19 to 43 with a mean and median age of 30. The average gestation period was 25 weeks, ranging from 10 weeks to 40 weeks. *Ureaplasma* species were detected in 76% (73/96) of specimens and 39.7% (29/73) of *Ureaplasma* positive specimens were also positive for *M. hominis. Mycoplasma hominis* was not detected alone. The colorimetric assay produced no discordant results; a slight colour change resulted in an orange colour (a positive result) and the content of none of the wells looked turbid, which was indicative of no contamination. To confirm results, all the trays were incubated for an additional 24 hours and reread. The growth of *Ureaplasma* species and *M. hominis* at various breakpoints of antimicrobial agents are displayed in Table 1. *Ureaplasma* species and *M. hominis* in a single specimen were noted as mixed isolates.

**Table 1:** **The distribution (%) of *Ureaplasma *** **species and *M. hominis *** **at different breakpoints of antimicrobial agents (n = 96)**

	**Levofloxacin**			**Moxifloxacin**		**Erythromycin**		**Clindamycin**		**Tetracycline**			
	**1**^**1**^	**2**	**4**	**0.25**	**2**	**8**	**16**	**0.25**	**0.5**	**1**	**2**	**4**	**8**
**Sensitive (S)/Resistant (R)**	**S**	**R**	**R**	**S**	**R**	**R**	**R**	**R**	**R**	**R**	**R**	**R**	**R**
***Ureaplasma*** **species (n = 44)**	52	30	11	93	2	70	9	0	100	25	16	2	30
***Ureaplasma *** **species and *M. hominis *** **(n = 29)**	55	28	14	83	10	10	86	0	100	14	28	7	48

^1^The breakpoints in μg/mL according to the CLSI guidelines [30].

**Figure 1:**
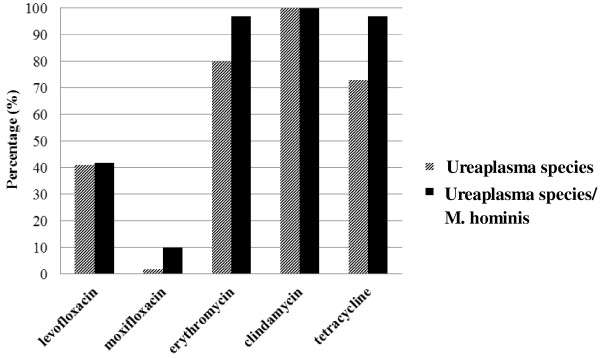
**Antimicrobial resistance (%) of *Ureaplasma *** **species and *M. hominis *** **positive specimens to various antimicrobial agents.**

Susceptibilities of 59% (26/44) and 98% (43/44) in the *Ureaplasma* species were found for levofloxacin and moxifloxacin respectively. Eighty percent (35/44) of *Ureaplasma* strains were resistant to erythromycin, whereas resistance to tetracycline was detected in 73% (32/44). Greater resistance was observed for mixed isolates against erythromycin and tetracycline [both 97% (28/29)] at higher critical concentrations. The susceptibility patterns of the mixed isolates to levofloxacin were similar to the cases where only *Ureaplasma* species were detected. Mixed isolates were more resistant to moxifloxacin (Figure 1).

The mPCR assay indicated that 95% (42/44) of the *Ureaplasma* positive specimens contained only *U. parvum,* while 5% (2/44) contained both *U. parvum* and *U. urealyticum*. None of the specimens that were positive for *Ureaplasma* species had only *U. urealyticum* present.

## Discussion

This study, together with a previously published article [27], is the first to report antimicrobial susceptibility patterns using the Mycofast Revolution assay from genital mycoplasmas in this specific setting. The high level of antimicrobial resistance to erythromycin and tetracycline that was observed for mixed isolates could be attributed to *M. hominis*. The antimicrobial resistance was significantly higher than when only *Ureaplasma* species were detected (97% vs 80% for erythromycin, p = 0.0396; and 97% vs. 73% for tetracycline, p = 0.0101).

Erythromycin resistance of mixed isolates is comparable to Domingues *et al*. [31] and Kechagia and colleagues’ [14] documentation of 90.7% and 100% erythromycin resistance respectively. Tetracycline resistance of mixed isolates was higher (97%) when compared to similar studies (14% to 57%) [14,22]. Resistance of *Ureaplasma* species to erythromycin (80%) is supported by some studies (83% [14]) but not by others (17.2% [22]). The susceptibility of *Ureaplasma* isolates to tetracycline was found to be 27%. Other studies have reported higher tetracycline susceptibility rates for *Ureaplasma* isolates, ranging from 81% to 100% [1,14,22]. Most of these studies reported lower tetracycline resistance rates specifically for the species *U. urealyticum*, which may explain the difference in resistance observed [1,14,31]. The demographics of the participants in the last-mentioned studies differed in the sense that one study reported on non-pregnant women of a wider age range (18–62) [14], while another reported on a group of pregnant women of gestation >20 weeks [1].

Results of the mPCR assay were that 95% (42/44) of the *Ureaplasma* positive specimens contained only *U. parvum,* while 5% (2/44) contained both *U. parvum* and *U. urealyticum. Ureaplasma parvum* was the principal species contributing to antimicrobial resistance. Similar results were found by Povlsen *et al*. [32], who investigated genital mycoplasmas in pregnant women with singleton pregnancies. These studies reported that approximately 90% of the 280 *Ureaplasma* positive specimens contained *U. parvum* and 3% contained both *U. parvum* and *U. urealyticum*. After speciation of the *Ureaplasma* positive specimens, *U. parvum* strains were discovered to confer resistance to fluoroquinolones (levofloxacin and moxifloxacin) and macrolides (erythromycin). These results are similar to those of Govender *et al*. [33], who reported fluoroquinolone and erythromycin resistance in *U. parvum* strains from South Africa.

Zhu and colleagues [34] reported antimicrobial susceptibilities of 10.65% and 31.27% to levofloxacin for mixed isolates and *U. urealyticum* respectively. The present study found genital mycoplasmas to be more susceptible to levofloxacin, with susceptibilities of 41% and 42% for *Ureaplasma* species and mixed isolates respectively.

The susceptibilities of genital mycoplasmas to antimicrobial agents differ by geographical region [22]. The difference in the antimicrobial resistance found in the present study and in reports from various countries might be the result of different antimicrobial-usage guidelines, which would lead to the resistance of bacterial strains to different antimicrobial groups [14]. Additional variables contributing to the difference in resistance may include the population studied, the study period, or the kits used for specimen processing and analyses [35]. The establishment of common guidelines for the treatment of genital mycoplasma infections is complex and effective treatment depends on the antimicrobial susceptibilities of genital mycoplasmas in a specific region [10,14].

Strategies to preserve the use of current antimicrobial agents and minimise resistance to such agents may include the development of: (i) new classes of antimicrobial agents; (ii) updated derivatives of currently used antimicrobial agents and; (iii) and the use of two different antimicrobial agents for treatment instead of one agent [36]. However, effective resources are required to constantly monitor antimicrobial susceptibility profiles of such agents to ensure treatment success and good pregnancy outcomes [10,36].

To circumvent the delays that may be experienced with the diagnosis of genital mycoplasma infections, the syndromic management of sexually transmitted infections (STIs) is a general treatment approach followed in South Africa. Patients are requested to report symptoms indicating the presence of an STI; e.g. vaginal discharge and/or dysuria and/or vulval itching or burning. In the present study, the symptoms that women reported were vague and subjective and did not correlate with results. The women could not definitely distinguish between a physiological discharge and an unusual vaginal discharge. Broad-spectrum antimicrobial regimens are used to cover all potential pathogens and commonly include combining a cephalosporin (e.g. cefixime) with erythromycin and metronidazole. A number of studies report that the syndromic approach is not effective in reducing the prevalence of curable STIs in asymptomatic patients [37-39]. Our study supports these findings by showing a very high percentage of resistance to erythromycin in this study group. Nonetheless, the success of the syndromic management of STIs relies on up-to-date knowledge of the infectious agents causing specific syndromes and the antimicrobial susceptibilities of these agents [40]. The frequent use of erythromycin in pregnant women has permitted the surveillance of long-term effects of this antimicrobial agent. These include infantile hypertrophic pyloric stenosis [41], cardiac toxicity [42] and maternal hepatotoxicity [43]. There is not yet enough data available to know whether the risks of toxicity in neonates are similar with newer macrolide antimicrobial agents [13]. If the price of azithromycin in many countries decreases to an affordable level, it may potentially replace erythromycin as a general treatment option in the future. Fluoroquinolones are classified as category C agents and the use of these agents in pregnancy is controversial [28]. The treatment options of genital mycoplasmas in pregnancy therefore remain limited.

Monitoring of susceptibility patterns of genital mycoplasmas may assist with optimising treatment guidelines and overall improve therapeutic outcomes. The Mycofast Revolution assay is an easy and effective way of evaluating the susceptibility of genital mycoplasmas to commonly used or potential antimicrobial agents [27].

A limitation of the study is that bacterial strains were not analysed for specific mutations where antimicrobial resistance was detected. Another limitation may be the inability of the authors to test for other pathogens, such as *Neisseria gonorrhoeae* and *Chlamydia trachomatis* and specifically for other *Mycoplasma* spp., such as *Mycoplasma genitalium*. No approved commercially available diagnostic assay exists for the detection and antimicrobial-resistance testing of all genital mycoplasma species, including *M. genitalium*. Detection of such species is mainly by nucleic acid amplification tests (NAATs) [44]. More studies in the Pretoria region are needed to confirm the high resistance rates of genital mycoplasmas to common antimicrobial agents and to determine the specific genetic elements responsible for resistance. These studies can be performed in broader study populations, such as non-pregnant women and HIV-positive women.

## Conclusion

Although the fluoroquinolones, especially moxifloxacin, remain the most effective against genital mycoplasmas, these agents are restricted to non-pregnant patients. A discrepancy in antimicrobial susceptibility in different regions emphasises the importance of routine monitoring to ensure the efficacy of treatment and curb morbidity and mortality rates.

## Competing interests

The authors declare no competing of interests.

## Authors’ contributions

MJR was involved in concept design, laboratory work as well as writing of the manuscript. MMK, MME and AWD were involved in concept design of the study as well as critical review of the manuscript. HAL was involved in concept design of the study as well as overseeing the logistics of sample collection. All authors read and approved the final manuscript.

## Pre-publication history

The pre-publication history for this paper can be accessed here:

http://www.biomedcentral.com/1471-2334/14/171/prepub
